# Supplementation of *Lactobacillus plantarum* ATCC14917 mitigates non-alcoholic fatty liver disease in high-fat-diet-fed rats

**DOI:** 10.3389/fmicb.2023.1146672

**Published:** 2023-05-17

**Authors:** Xingjian Wen, Hejing Liu, Xiaoling Luo, Li Lui, Jiuyu Fan, Yajing Xing, Jia Wang, Xingfang Qiao, Na Li, Guixue Wang

**Affiliations:** ^1^Chongqing Academy of Chinese Materia Medica, Chongqing, China; ^2^College of Bioengineering, Chongqing University, Chongqing, China; ^3^Clinical College of Traditional Chinese Medicine, Hubei University of Chinese Medicine, Wuhan, China

**Keywords:** non-alcoholic fatty liver disease, *Lactobacillus plantarum* ATCC14917, oxidative stress, inflammation, gut microbiota

## Abstract

Atherosclerosis and non-alcoholic fatty liver disease (NAFLD) have been increasing at an alarming rate worldwide. Many clinical studies have underlined the link between NAFLD and atherosclerosis. Our previous experiments have discovered that *Lactobacillus* (L.) *plantarum* ATCC14917 supplementation could decrease the progression of atherosclerotic lesion formation. In this study, we aimed to investigate the role of supplementation of *L. plantarum* ATCC14917 mitigates liver injury in rats fed with a high-fat diet (HFD, 45% kcal from fat). A total of 32 rats were randomly divided into four groups, including two intervention groups, who fed with HFD and administering either 1 × 10^7^ or 1 × 10^9^ colony forming units (CFU) of *L. plantarum* ATCC14917, the normal control group, and the HFD control group. The results showed that supplementation with low-dose and high-dose of *L. plantarum* ATCC14917 for 8 weeks could alleviate the body weight gain (*p* < 0.05), hepatic steatosis, and serum lipid metabolism (*p* < 0.05) in HFD-fed rats. Moreover, supplementation of *L. plantarum* ATCC 14917 decreased total cholesterol (TC), triglyceride (TG), alanine aminotransferase (ALT), and aspartate aminotransferase (AST) levels (*p* < 0.05) in serum, and improved HFD-associated inflammation (*p* < 0.05). Furthermore, cecal contents were analyzed by high-throughput 16S ribosomal RNA sequencing. The results indicated that supplementation of *L. plantarum* ATCC 14917 could ameliorate HFD-induced gut dysbiosis. In summary, our findings suggest that supplementation of *L. plantarum* ATCC 14917 could mitigate NAFLD in rats, suggesting it may be considered as a probiotic agent for preventing HFD-induced obesity.

## 1. Introduction

Non-alcoholic fatty liver disease (NAFLD) is one of the most common chronic liver diseases, characterized by excessive lipid deposition and steatosis in liver cells. It is closely related to metabolic syndromes such as obesity, insulin resistance, dyslipidemia, and hypertension. NAFLD is a clinicopathological syndrome, ranging from non-alcoholic fatty liver (NAFL) to non-alcoholic steatohepatitis (NASH), which can progress to cirrhosis and liver cancer (Kim et al., 2021; Lange et al., 2021). The pathogenesis of NAFLD has not been fully elucidated, and the pathophysiology is related to multiple simultaneous factors. The high-fat diet (HFD) can cause oxidative stress and lipid peroxidation in the liver, leading to internal fat deposition in hepatocytes, resulting in the formation of NAFLD (Friedman et al., 2018; Du et al., 2020). The intake of HFD can cause an imbalance of the gut microbiota (Liang et al., 2021), which leads to the excessive reproduction of harmful bacteria in the intestine, leading to the increased generation of intestinal endotoxin and the imbalance of the gut barrier stability (Gil-Gomez et al., 2021). The microbiota-generated metabolites and other compounds could enter the liver through the portal vein, which activates Toll-like receptors and emit a signal transduction cascade to release cytokines and chemokines, causing inflammatory responses, oxidative stress, and lipid peroxidation (Grabherr et al., 2019; Ferro et al., 2020; Khan et al., 2021).

At present, there is a lack of specific drugs for the clinical treatment of NAFLD. Although there are some drugs with hypolipidemic effects that can improve the symptoms of NAFLD, they may be accompanied by adverse side effects (Romero-Gomez et al., 2017; Neuschwander-Tetri, 2020). Increasing numbers of studies have demonstrated that probiotics can effectively reduce endotoxemia, improve gut barrier function, and may play a certain role in the treatment of NAFLD (Wang et al., 2020; Ferguson and Finck, 2021; Luo et al., 2021; Ren et al., 2021; Huang et al., 2022). As the most studied probiotic bacteria, *Lactobacillus* is an important member of the maintenance of microbial flora in the gut, and some strains have been widely used in the field of food and health and have been developed as probiotics (Liang et al., 2020; Zhang et al., 2020; Lee et al., 2021; Yu et al., 2021; Zhu et al., 2021).

Our previous studies have shown that *Lactobacillus (L.) plantarum* ATCC14917 could alleviate the progression of atherosclerotic lesion formation in mice, and can significantly improve inflammatory and oxidative stress (Hassan et al., 2020). But the effect of *L. plantarum* ATCC14917 on NAFLD has not been elucidated. Therefore, we investigated the effects of *L. plantarum* ATCC14917 on HFD-induced NAFLD in this study. The rat model of NAFLD induced by HFD was intervened with *L. plantarum* ATCC14917 for 8 weeks. The ability of *L. plantarum* ATCC14917 on alleviating NAFLD was assessed by the changes in lipid deposition, oxidative stress, inflammation, and gut microbiota diversity and compositions. This study aimed to provide a basis and reference for the application of *L. plantarum* ATCC14917 in the intervention of NAFLD.

## 2. Materials and methods

### 2.1. Materials and reagents

The phosphate-buffered saline (PBS, 022117) and de Man-Rogosa-Sharpe (MRS, 027315) broth were purchased from HKM Company (Guangdong, China). The animal chow was purchased from Jiangsu Xietong Pharmaceutical Bio-Engineering Co., Ltd. (Jiangsu, China), including HF-diet (45% kcal from fat, XTHF45 according to the Research Diet D12451) and a matched control diet (10% kcal from fat, XTCON50H). The hematoxylin and eosin (HE) kit (G1001), picrosirius red staining kit (GC307014), and Oil red O staining kit (G1015) were purchased from Servicebio technology Co., Ltd. (Wuhan, China). Serum levels of the triglyceride (TG, 100045051416), total cholesterol (TC, 100045051438), alanine aminotransferase (ALT, 100045051446), and aspartate aminotransferase (AST, 100045051470) were measured by TG assay kit (GPO-PAP), TG assay kit (COD-PAP), ALT and AST assay kit (IFCC) (Shenzhen Mindray Bio-Medical Electronics Co., Ltd., Shenzhen, China) using an automatic biochemical detector BS-240VET (Shenzhen Mindray Bio-Medical Electronics Co., Ltd., Shenzhen, China). The ELISA kits of tumor necrosis factor (TNF-α, MM-0180R1), interleukin-1β (IL-1β, MM-0047R1), and interleukin-6 (IL-6, MM-0190R1) were purchased from Jiangsu Enzyme Immunity Industry Co., Ltd. (Jiangsu, China). The superoxide dismutase(SOD) activity assay kit (spectrophotometer, BC0170), malondialdehyde (MDA) content assay kit (spectrophotometer, BC0025), and glutathione peroxidase (GSH-Px) activity assay kit (spectrophotometer, BC1195), chromogenic end-point TAL assay kit (T7574) was purchased from Solarbio Science & Technology Co., Ltd. (Beijing, China). Other reagents and chemicals used in this investigation were purchased from Aladdin Chemistry Co., Ltd. (Shanghai, China) and were of analytical grade.

### 2.2. Preparation of the bacterial solution

*L. plantarum* ATCC 14917 was purchased from China General Microbiological Culture Collection Centre (www.cgmcc.net). L. *plantarum* ATCC 14917 was cultured anaerobically in MRS broth at 37°C for 16–18 h. After removing the supernatant by centrifugation (3,500 × g, 10 min, 4°C), the cells were washed with sterile PBS two times and resuspended in PBS. The suspension concentration of *L. plantarum* ATCC 14917 was adjusted to approximately 1 × 10^7^ or 1 × 10^9^ colony-forming units of bacteria per mL (CFU/mL) to prepare for the gavage of rats as described previously (Hassan et al., 2020).

### 2.3. Animals experimental design

A total of 32 specific-pathogen-free (SPF) male Sprague Dawley rats (6 weeks old, 180–200 g) were purchased from Hunan Silaikejingda Experimental Animal Company Limited (Changsha, Hunan, China). Rats were kept under controlled environmental conditions at 25 ± 1°C with a 12 h light/dark cycle and access to food and water freely. After acclimation for 1 week, rats were then randomly divided into the following four experimental groups (*n* = 8/group): the (1) control diet group (CON); (2) high-fat diet group (HFD); (3) high-fat diet with *L. plantarum* ATCC 14917 (1 × 10^8^ CFU/ml) supplement group (HFDLP1); (4) high-fat diet and *L. plantarum* ATCC 14917 (1 × 10^9^ CFU/ml) supplement group (HFDLP2). The rats in the CON and HFD groups received equivalent PBS (2 ml) daily through oral gavage for 8 weeks. During the experiments, the body weight of rats was measured every week. After the last administration, the rats were fasting for 12 h but drank water freely. The rats were sacrificed after euthanasia at the end of the experiment. Then the blood, liver tissues, ileum tissues, and colonic content samples of rats were taken for further testing.

### 2.4. Serum biochemical analysis

Following anesthetization, the blood was collected and coagulated naturally at room temperature for 10 min. Afterward, serum samples were collected after centrifugation for 20 min (3,000 × g at 4°C) and stored at −80°C until use. The levels of ALT, AST, TG, and TC in the serum were detected by an automatic biochemical analyzer (Mindray BS-240VET, China). The lipopolysaccharides (LPS) concentration in serum was detected with the chromogenic end-point TAL assay kit according to the manufacturer’s instructions. The ELISA double-antibody sandwich method was used to detect the levels of proinflammatory cytokines IL-1β, IL-6, and TNF-α in the serum of rats in each group. All the operation method is carried out by the instructions of the purchased kit.

### 2.5. Histological and staining analysis

Partial liver and ileum tissues were cleaned with ice-cold PBS. Then the tissues were fixed with 10% neutral formalin for 24 h, dehydrated with ethanol, dealcoholized with xylene, and then embedded in paraffin. Subsequently, the liver tissues were sectioned into 4-μm-thick slices. The liver sections of each group of rats were stained with HE or Picrosirius red after deparaffinization, according to the kit manufacturer. The pathological images were acquired with a light microscope (Leica DM 2500, Germany) at 200× magnifications. Meanwhile, Oil red O staining was performed to observe liver intracellular lipid accumulation. Five fields of vision in each sample were collected at random.

### 2.6. Determination of hepatic MDA, GSH-PX, and SOD levels

The liver samples were thoroughly rinsed in PBS and then homogenized in PBS in a ratio of 1:10 (w/v) for the detection of antioxidant biomarkers (MDA, GSH-PX, and SOD). The levels of MDA, GSH-PX, and SOD in the supernatant of homogenates were detected by using commercial kits following the manufacturer’s protocol, respectively.

### 2.7. RNA extraction and reverse transcription real-time PCR

The TLR-4, MyD88, and NF-κB gene mRNA expressions were analyzed by real-time quantitative PCR (RT-qPCR). Total RNA was isolated from 50 mg of rat liver tissue using TRIzol Reagent (15596026, Invitrogen, United States) following the manufacturer’s instructions. Isolated RNA was then quantified (1 μg) and reverse transcribed into cDNA with the cDNA Reverse Transcription Kit (4368813, Invitrogen, United States) according to the manufacturer’s instructions. The cDNA was then diluted at 1:50 in RNase-free water and kept at −20°C until further use. qPCR analysis was carried out in 96-well plates with a BioRad CFX-96 real time system (BioRad, United States) using SYBR Premix Ex Taq II (RR820A, TaKaRa, Japan). Gene-specific primer sequences used are shown in Table 1. The amplification conditions were denaturation at 95°C for 10 min, followed by 40 cycles of 95°C for 15 s and extension at 60°C for 30 s. The relative levels of the target genes were normalized by the expression of glyceraldehyde-3-phosphate dehydrogenase (GAPDH). Quantitative changes in gene expression were quantified using the 2^–ΔΔCT^ method.

**Table 1:** Real-time quantitative PCR primers used in analyses of liver tissues.

Target gene	Primer	Sequences (5 → 3)
TLR4	Forward	GATTGCTCAGACATGGCAGTTTC
	Reverse	CACTCGAGGTAGGTGTTTCTGCTAA
MyD88	Forward	AGGAGGACTGCCAGAAATACATAC
	Reverse	GATGCCTCCCAGTTCCTTTG
NF-κB	Forward	AGGAAGGCAAAGCGAATCCA
	Reverse	TCAGAACCAAGAAGGACGCC
GAPDH	Forward	GAAGGTCGGTGTGAACGGATTTG
	Reverse	CATGTAGACCATGTAGTTGAGGTCA

### 2.8. 16S rRNA gene sequencing of the gut microbiota

The colonic content samples were snap-frozen and stored in liquid nitrogen. Total microbiota genomic DNA from these samples was extracted with biomarker soil genomic DNA kit (RK02008, Beijing Biomarker Technologies Co. Ltd., Beijing, China), and used for 16S rRNA gene analysis for microbiota profiling with barcoded amplicons from the V3–V4 region of the 16S rRNA gene as described previously (Park et al., 2020; Wang et al., 2021). The V3-V4 region of the 16S rRNA gene was amplified by PCR using the primer pair 338F (forward, 5′-ACTCCTACGGGAGGCAGCAG-3′) and 806R (reverse, 5′-GGACTACHVGGGTWTCTAAT-3′). The purified amplicons were pooled and sequenced by the Illumina Novaseq 6000 system (Illumina, San Diego, United States) according to the standard protocols by BioCloud biotechnology company (Shanghai, China). Raw reads were generated and analyzed using the BioCloud platform^1^. The raw sequence data were deposited to the National Center for Biotechnology Information with the following accession number: PRJNA892456. The alpha diversity and beta diversity analyses were performed based on OTU clustering. In addition, the changes in gut microbiota at the phylum level were analyzed as our described previously (Li et al., 2023).

### 2.9. Statistical analysis

Data from these experiments were presented as mean ± standard deviation (SD). Data were analyzed with the GraphPad Prism software version 9.0 (San Diego, CA, United States). The statistical analysis (unpaired parametric *t*-test) was performed. A probability (*P*) value less than 0.05 indicates statistical significance.

## 3. Results

### 3.1. Body weight and serum lipid levels

**Figure 1:**
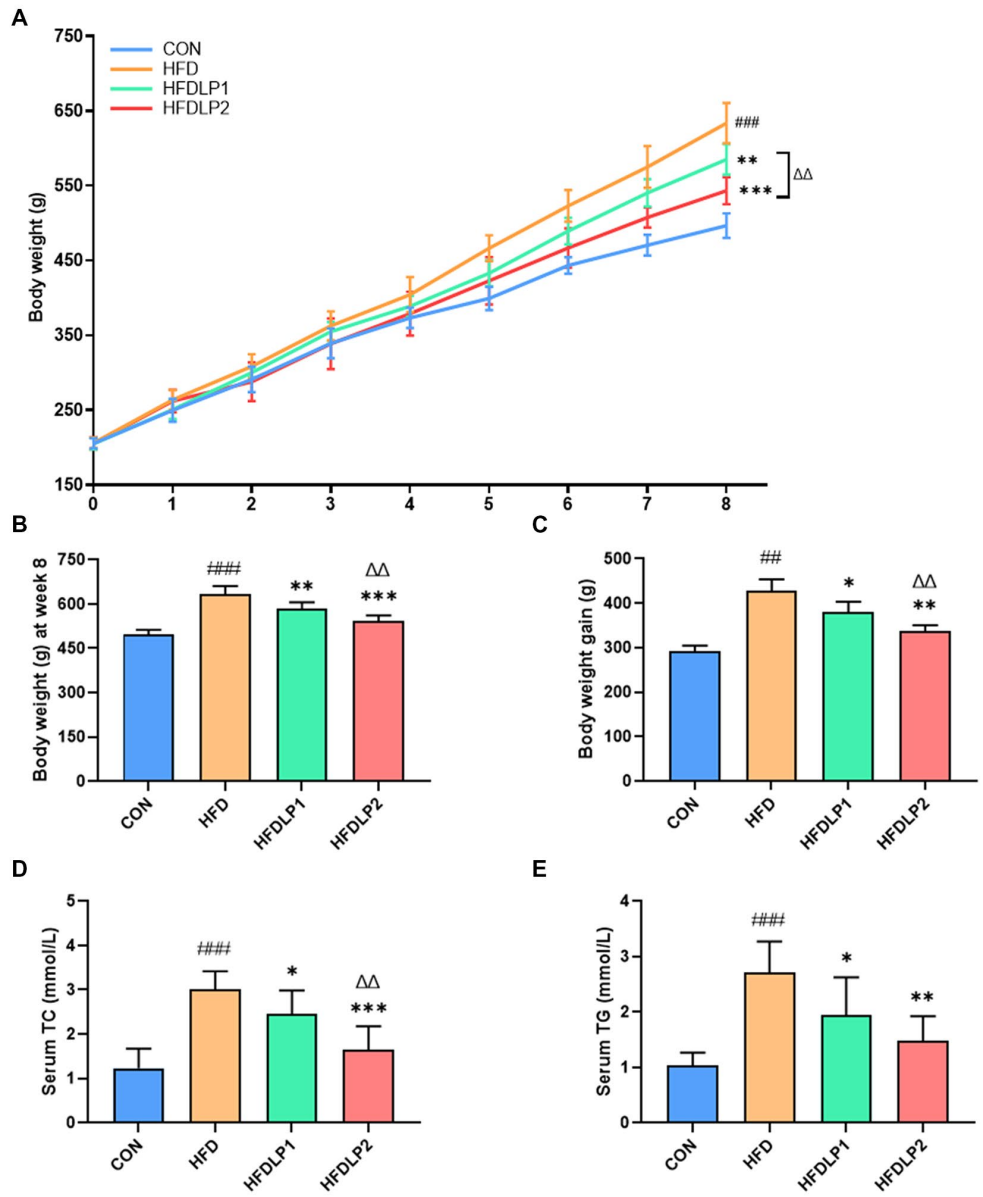
Effects of *Lactobacillus plantarum* ATCC14917 on body weight and lipid metabolism in rats. **(A)** The body weight changes in rats for 8 consecutive weeks; **(B)** the body weight of rats after 8 weeks of feeding; **(C)** the body weight gain of rats after 8 weeks of feeding; **(D)** the serum TC; **(E)** the serum TG. Data are presented as means ± SD (*n* = 8) and analyzed using the *t*-tests. ^##^*p* < 0.01 and ^###^*p* < 0.005 compared with the CON group. ^*^p < 0.05, ^**^p < 0.01, ^***^p < 0.005 compared with the HFD group. ^△△^*p* < 0.01 compared with the HFDLP1 group.

A Comparison of rat body weight in each group is shown in Figure 1A. At the beginning of the experiment (weeks 1–4), there was no significant difference in the body weight between different groups (*p* > 0.05). The body weight of rats changed significantly after 5–8 weeks of treatment under different intervention conditions. After 8 weeks of feeding, the body weight in rats of the HFD group was significantly higher than that of the CON group (*p <* 0.005, Figure 1B). The body weight of rats in the HFDLP1 and HFDLP2 groups was significantly lower than the HFD group (*p* < 0.01). Meanwhile, the body weight gain in the HFDLP1 and HFDLP2 groups was significantly lower than in the HFD group (*p* < 0.05, Figure 1C). In addition, the levels of serum TC and TG in rats of the HFD group increased significantly compared with the normal group (*p* < 0.001, Figures 1D,E). Compared with the HFD group, serum TC and TG in rats in the HFDLP1 group were significantly reduced (*p* < 0.05), the levels of serum TC in rats of the HFDLP2 group were significantly reduced (*p* < 0.01), and TG of rats in the HFDLP2 group were also significantly reduced (*p* < 0.01). These results indicate that supplementation of *L. plantarum* ATCC14917 could regulate the body weight gain and serum lipid levels of rats induced by HFD.

### 3.2. Pathological analysis

**Figure 2:**
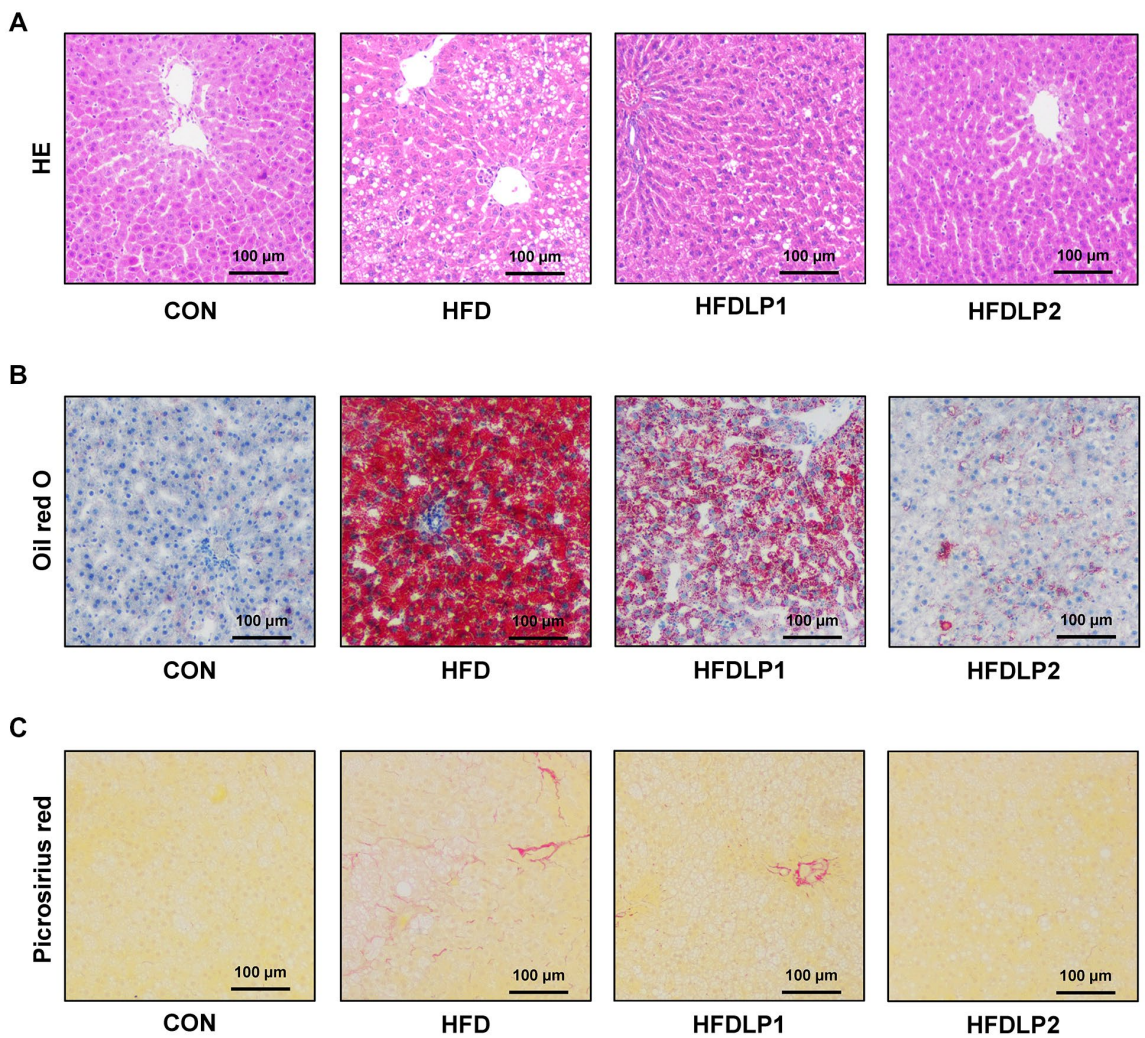
Effects of *Lactobacillus plantarum* ATCC14917 on liver injury, hepatic steatosis, and fibrosis in rats. **(A)** The HE staining of liver tissue sections; **(B)** the Oil red O staining of liver tissue sections; **(C)** the Picrosirius red staining of liver tissue sections. *n* = 5, at 200× magnifications.

The effects of *L. plantarum* ATCC14917 on the pathological changes of the liver were shown in Figure 2. The results of HE staining showed that the liver tissue of the CON group had no obvious pathological changes. In contrast, the liver tissue of the HFD group showed classical pathophysiological characteristics of hepatic steatosis. Hepatocytes were swollen and sparsely arranged, with many lipid droplet vacuoles in the cytoplasm, nuclei moved to the edge, and inflammatory cells were infiltrated in some areas of the tissue in HFD-fed rats. Meanwhile, the results of Oil Red O staining revealed marked lipid droplet deposition. Compared with the HFD group, the pathological changes in the liver tissue of the HFDLP1 and HFDLP2 groups were alleviated. Especially, the results of Oil Red O staining and Picrosirius red staining proved that high-dose supplementation with *L. plantarum* ATCC14917 reduced liver lipid accumulation and fibrosis in HFD-fed rats.

### 3.3. Serum biochemical parameters and oxidative stress levels

**Figure 3:**
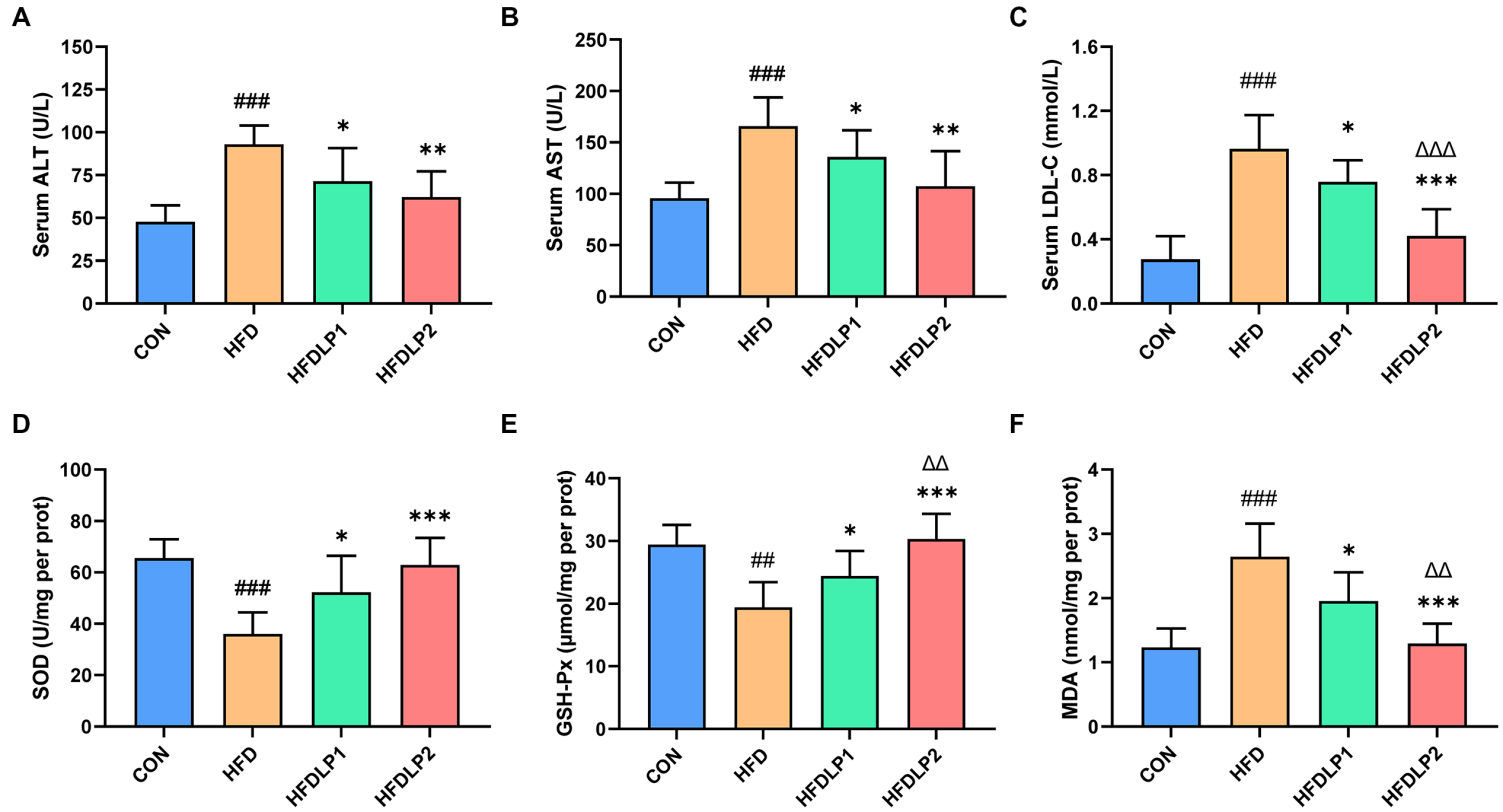
Effects of *Lactobacillus plantarum* ATCC14917 on serum biochemical parameters and oxidative stress levels. **(A)** The serum ALT; **(B)** the serum AST; **(C)** the serum LDL-C; **(D)** the liver SOD; **(E)** the liver GSH-PX; **(F)** the liver MDA. Data are presented as means ± SD (*n* = 8) and analyzed using the *t*-tests. ^##^*p* < 0.01 and ^###^*p* < 0.005 compared with the CON group. ^*^p < 0.05, ^**^p < 0.01, ^***^p < 0.005 compared with the HFD group. ^△△^*p* < 0.01 and ^△△△^*p* < 0.005 compared with the HFDLP1 group.

The levels of ALT, AST, SOD, GSH-PX, and MDA in serum or liver were measured to evaluate the effect of *L. plantarum* ATCC14917 on liver function and oxidative stress. As shown in Figure 3, the levels of serum ALT and AST in the HFD group were significantly higher than those in the CON group (*p* < 0.01). In addition, the levels of serum AST and ALT in the HFDLP1 and HFDLP2 groups were significantly lower than that in the HFD group (*p* < 0.05). These results suggested that *L. plantarum* ATCC14917 could improve liver injury. Furthermore, the results showed that the activity of SOD and the content of GSH-Px in the liver were decreased with HFD, but supplementation with *L. plantarum* ATCC14917 significantly increased these levels (*p* < 0.05). In contrast, the content of MDA significantly increased in the HFD group compared with the CON group. Meanwhile, the supplementation with *L. plantarum* ATCC14917 effectively inhibited the upregulation of MDA content in the liver compared to the HFD group.

### 3.4. The levels of inflammatory cytokines and representative expression of TLR4/NF-κB signaling pathway

**Figure 4:**
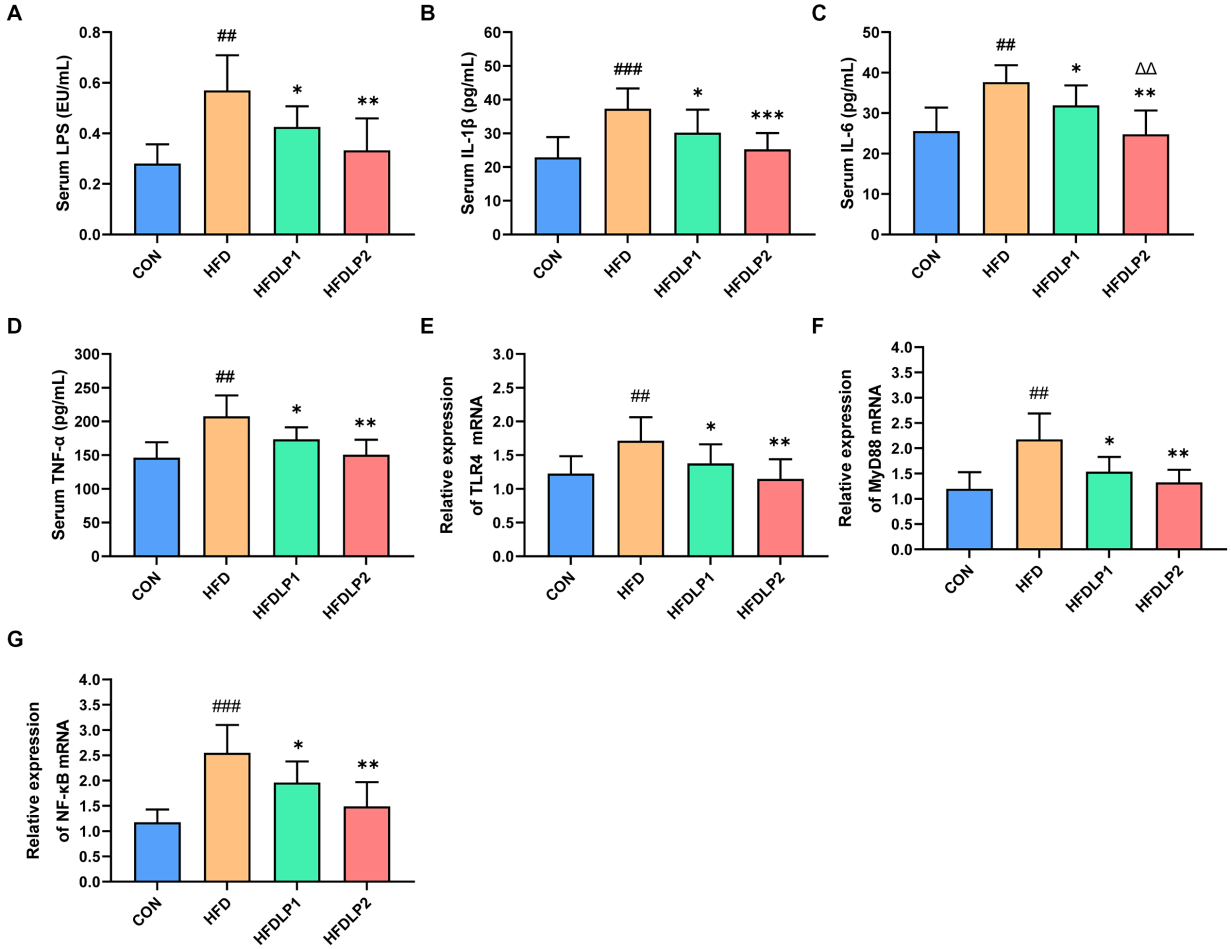
Effects of *L. plantarum* ATCC14917 on the levels of LPS and inflammatory cytokines in serum, and representative expression of TLR4/NF-κB signaling pathway in the liver. **(A)** serum LPS; **(B)** serum IL-1β; **(C)** serum IL-6; **(D)** serum TNF-α; **(E)** the mRNA expression levels of TLR4; **(F)** the mRNA expression levels of MyD88; **(G)** the mRNA expression levels of NF-κB. Data are presented as means ± SD (*n* = 8) and analyzed using the *t*-tests. ^##^*p* < 0.01 and ^###^*p* < 0.005 compared with the CON group. ^*^p < 0.05, ^**^p < 0.01, ^***^p < 0.005 compared with the HFD group; ^△△^*p* < 0.01 compared with the HFDLP1 group.

Accumulating evidence suggested that HFD could increase LPS concentration in serum, which plays an important role in NAFLD progression. Therefore, we examined the level of LPS in serum with chromogenic end-point TAL assay. As shown in Figure 4A, serum LPS in the HFD group was significantly higher than that in the CON group (*p* < 0.05). Interestingly, the level of serum LPS in the HFDLP1 and HFDLP2 groups were both significantly lower than that in the HFD group (*p* < 0.05). To further explore the effect of *L. plantarum* ATCC14917 on inflammation in rats, we evaluated the changes in the content levels of IL-1β, IL-6, and TNF-α in the serum. As shown in Figures 4B–D, compared with the CON group, the levels of IL-1β, IL-6, and TNF-α in the HFD group were significantly increased (*p* < 0.01). With *L. plantarum* ATCC14917 intervention, the levels of these inflammatory cytokines were significantly reversed in the HFDLP1 and HFDLP2 groups, and no obvious difference was found compared with the CON group.

To further explore the effect of *L. plantarum* ATCC14917 on the TLR4/NF-κB signaling pathway, we assessed the mRNA expression levels of TLR4, MyD88, and NF-κB in the liver. The results were listed in Figures 4E–G. Compared with the CON group, the mRNA expression levels of TLR4, MyD88, and NF-κB were significantly increased in the liver of the rats in the HFD group (*p* < 0.01). Besides, the mRNA expression levels of TLR4, MyD88, and NF-κB in the HFDLP1 and HFDLP2 groups showed significant downregulation in the liver compared to the HFD group (*p* < 0.05).

### 3.5. Gut microbiota diversity and compositions

**Figure 5:**
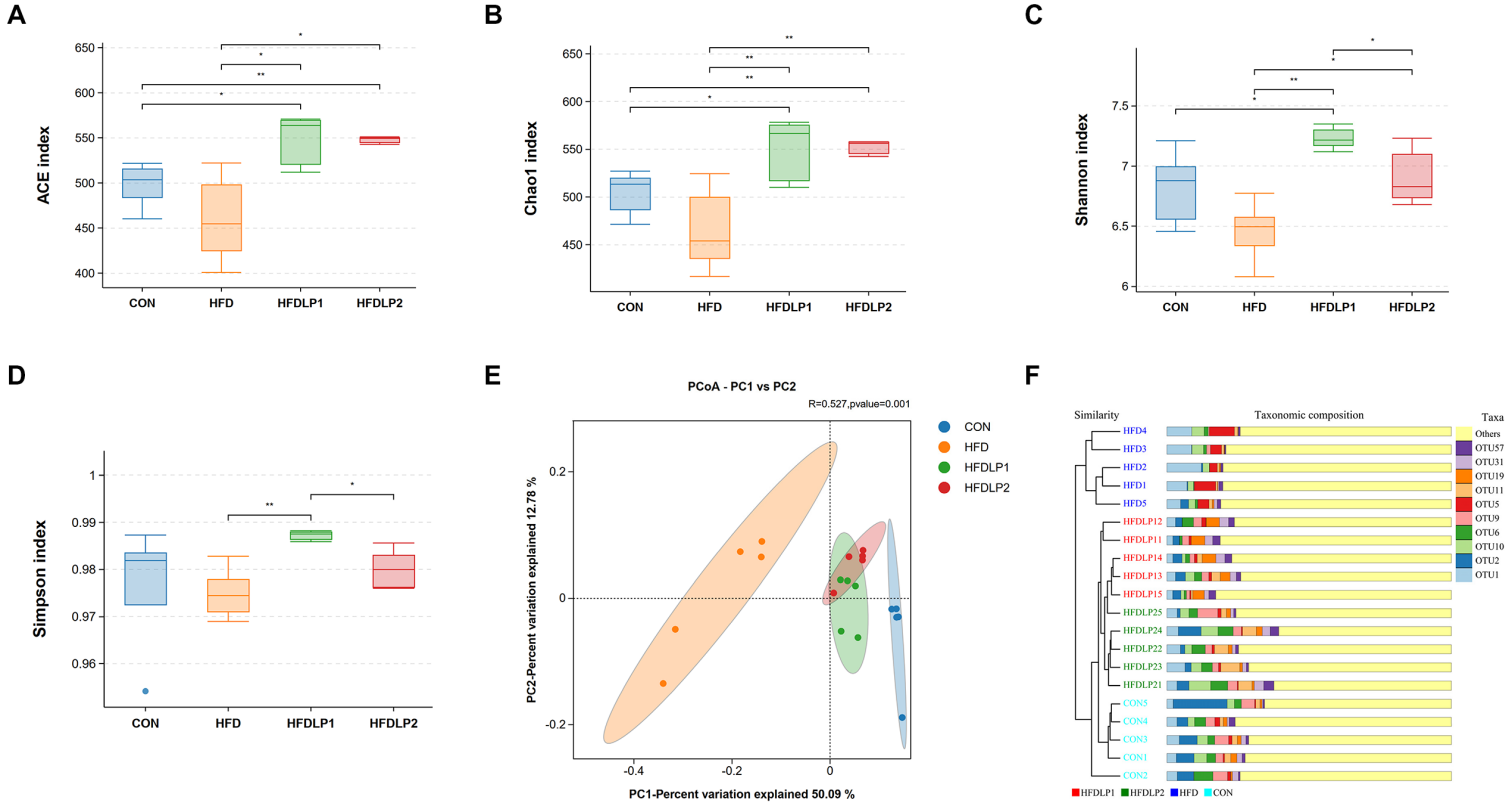
The alpha diversity and beta diversity analysis of the gut microbiota. **(A)** Simpson index; **(B)** Chao1 index; **(C)** Shannon index; **(D)** ACE index; **(E)** Principal co-ordinates analysis (PCoA); **(F)** Unweighted Pair-group Method with Arithmetic Means (UPGMA) analysis. Data are presented as means ± SD (*n* = 5) and analyzed using the *t*-tests. ^*^p < 0.05 and ^**^p < 0.01 compared with the other group.

To assess the beneficial effects on the gut microbiota of *L. plantarum* ATCC14917 supplementation, we measured the changes in gut microbiota by 16S rRNA gene sequencing. The alpha diversity and beta diversity analysis results of the cecal microbiota are shown in Figure 5. HFD induced a decrease in the species richness and diversity of gut microbiota compared with the CON group (*p* > 0.05). Noteworthily, supplementation of *L. plantarum* ATCC14917 increased the observed species richness (Chao1 and ACE) of gut microbiota compared with the HFD group (*p* < 0.05). Meanwhile, species diversity (Shannon) of gut microbiota also increased (*p* < 0.05) in the HFDLP1 and HFDLP2 groups. In addition, the results of the PCoA plot and UPGMA showed that the gut bacteria compositions of the HFDLP1 and HFDLP2 groups were distinctly separated from the HFD group (Figures 5E,F).

**Figure 6:**
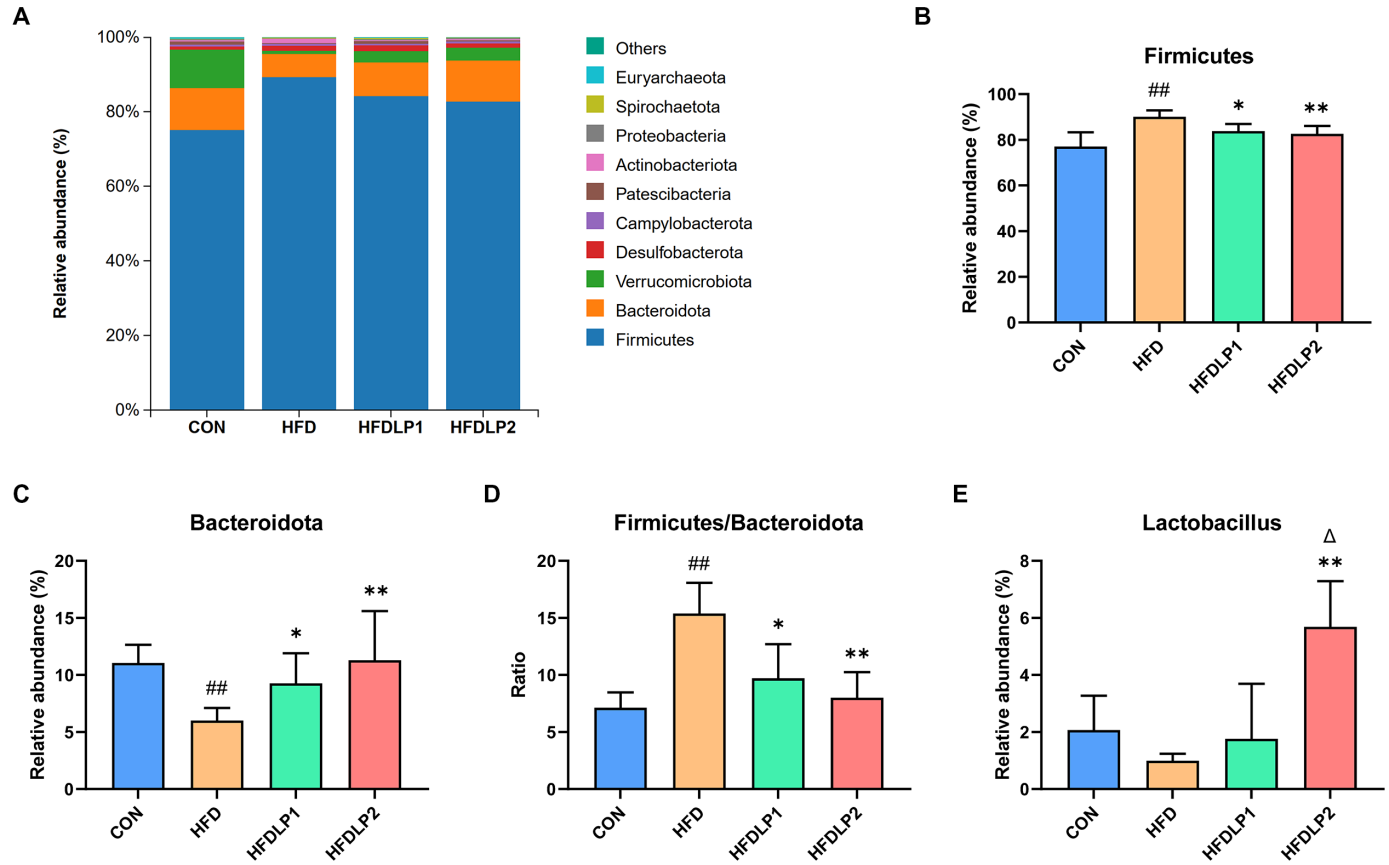
The changes in the microbiome composition. **(A)** The taxonomic composition distribution of relative abundance at the phylum level; **(B)** differences in the relative abundance of *Firmicutes*; **(C)** differences in the relative abundance of *Bacteroidota*; **(D)** differences in the ratios of *Firmicutes* to *Bacteroidetes* (F/B); **(E)** differences in the relative abundance of *Lactobacillus* at the genus level. Data are presented as means ± SD (*n* = 5) and analyzed using the *t*-tests. ^##^*p* < 0.01 compared with the CON group. ^*^p < 0.05 and ^**^p < 0.01 compared with the HFD group. ^△^*p* < 0.05 compared with the HFDLP1 group.

The changes in the microbiome composition between different treatment groups were apparent. As shown in Figure 6, the phylum-level taxonomic composition analysis showed that *Firmicutes* and *Bacteroidota* were the predominant bacterial phyla. HFD increased the relative abundances of *Firmicutes* and decreased the relative abundances of the *Bacteroidota* compared with the CON group (*p* < 0.01). In addition, the ratio of *Firmicutes* to *Bacteroidetes* (F/B) was up-regulated by HFD (*p* < 0.01). It was of interest that the ratios of F/B in the HFDLP1 and HFDLP2 groups were lower compared to the HFD group (*p* < 0.05). These results on the ratios of F/B were consistent with previous reports (Leung et al., 2016; Magne et al., 2020; Zhao et al., 2021; Di Ciaula et al., 2022), indicating that supplementation of *L. plantarum* ATCC14917 can reverse the changes in the structure of the gut microbiota induced by the HFD (Hassan et al., 2020). Specifically, the relative abundance of *Lactobacillus* in the HFDLP1 and HFDLP2 groups was higher than that in the HFD group.

## 4. Discussion

NAFLD is one of the most common clinical liver diseases in recent years, which can progress to liver fibrosis and cirrhosis, even liver cancer, and increase the risk of diabetes and cardiovascular disease (Sheka et al., 2020). Many clinical studies have underlined the link between NAFLD and atherosclerosis (Li et al., 2021; Tang et al., 2022). Patients with atherosclerosis are often accompanied by NAFLD (Stols-Goncalves et al., 2019). Our previous experiments have discovered that *L. plantarum* ATCC14917 supplementation could decrease the progression of atherosclerotic lesion formation (Hassan et al., 2020). Therefore, the objective of the present study is to investigate the role of supplementation of *L. plantarum* ATCC14917 on the NAFLD rats induced by HFD. The results showed that supplementation with low-dose and high-dose of *L. plantarum* ATCC14917 both alleviated the body weight gain, hepatic steatosis, and serum lipid metabolism in HFD-fed rats. Moreover, supplementation of *L. plantarum* ATCC 14917 decreased TC, TG, ALT, and AST levels in serum, and improved HFD-associated inflammation. These data revealed that *L. plantarum* ATCC14917 could drastically alleviate HFD-induced obesity and liver lipid deposition. In addition, the result of *L. plantarum* ATCC14917 supplementation on liver oxidative stress in HFD-fed rats was also consistent with previous report (Hassan et al., 2020). The *L. plantarum* ATCC14917 intervention increased the activity of SOD and the content of GSH-Px in the liver and inhibited the upregulation of MDA content in the liver of HFD-fed rats.

Previous studies have found that innate immunity plays an important role in the pathogenesis of NAFLD, including promoting and regulating key processes in the occurrence and development of NAFLD. Numerous studies have investigated the association between the LPS-TLR4-NF-κB signaling pathway with the pathogenesis of NAFLD (Bao et al., 2020). LPS plays a key role in gut microbiota changes, inflammation, and metabolic disorders (Carpino et al., 2020). Animal studies have shown that dysregulation of the gut microbiota caused by obesity impairs the integrity of the gut barrier, causing the release of LPS from intestinal gram-negative bacteria and subsequent LPS into mesenteric veins, triggering obesity-associated chronic inflammation by activating the TLR4/NF-κB signaling pathway (Wu et al., 2019; Guo et al., 2021; Xia et al., 2022). In our present study, we found that *L. plantarum* ATCC 14917 can significantly reduce the levels of LPS and inflammatory cytokines (IL-1β, IL-6, and TNF-α) in the serum of NAFLD rats, and downregulate the mRNA expression levels of TLR4, MyD88, and NF-κB in the liver. These results revealed that *L. plantarum* ATCC14917 could alleviate HFD-induced inflammation through the LPS-TLR4-NF-κB signaling pathway. Therefore, we hypothesized that *L. plantarum* ATCC14917 intervention may improve the inflammatory dysfunction of HFD-fed rats through the “gut-liver axis” pathway.

Studies have shown that there is a close link between the inflammatory response and the gut microbiota (Jiang et al., 2020; Li et al., 2022; Zhang et al., 2022). Altered gut microbiota can mitigate or promote inflammatory processes (Jiang et al., 2020; Pan et al., 2022). The inflammatory response and altered autoimmune status caused by gut microbiota play an important role in NAFLD pathogenesis. The gut microbiota affects the pathogenesis of NAFLD mainly through the following ways, including affecting intestinal permeability, energy absorption, sugar, and lipid and bile acid metabolism, regulating the expression of genes related to its related signaling pathways, and participating in the regulation of host immunity (Ji et al., 2019; Behary et al., 2021). In the intestine of rats on a normal diet, *Firmicutes* and *Bacteroidetes* are the dominant phyla (Gómez-Zorita et al., 2019). The HFD-fed changed the gut microbiota diversity and compositions in rats, especially reducing the abundance of *Bacteroidetes* and increasing the abundance of *Firmicutes*. The results of this study were consistent with the above study (Hassan et al., 2020), *L. plantarum* ATCC14917 can reverse the changes in the structure of the gut microbiota induced by the HFD, notably the major bacterial phylum. In recent years, many studies have shown that probiotics can regulate intestinal flora to prevent intestinal flora imbalance, improve intestinal barrier function, reduce oxidative stress and inflammation, and improve lipid metabolism and NAFLD (Cao et al., 2022; Hu et al., 2022; Arellano-García et al., 2023; Shin et al., 2023; Wang et al., 2023; Zhao et al., 2023). In addition, there are several studies on the effects of different probiotics on improving NAFLD in clinical trials (Arellano-García et al., 2022; Noormohammadi et al., 2023; Zhou et al., 2023). Probiotic/synbiotic supplementation have a good regulating effect on liver function.

## 5. Conclusion

In summary, *L. plantarum* ATCC14917 can mitigate NAFLD from lipid deposition, oxidative stress, the HFD-associated inflammation, and partly modulated the gut microbial composition and structure in rats, suggesting it may be an alternative therapy for the intervention of NAFLD.

## Data availability statement

The data presented in the study are deposited in the NCBI repository, accession number PRJNA892456.

## Ethics statement

The animal study was reviewed and approved by Ethics Committee of the Chongqing Academy of Chinese Materia Medica.

## Author contributions

XW, NL, and GW designed and interpreted experiments and wrote the manuscript. XW, HL, XL, and NL performed experiments and analyzed data. LL, JF, YX, JW, XQ, and GW provided intellectual inputs, contributed to the data collection and critically reviewed the manuscript. All authors contributed to the article and approved the submitted version.

## Funding

This research was supported by funds from the Chongqing Science and Technology Bureau, China (cstc2020jxjl-jbky10002, cstc2020jxjl-jbky0018, cstc2023jxjl-jbky0004, cstc2023jxjl-jbky0014, cstc2021jscx-dxwtB0007, and cstc2021jscx-dxwtBX0013), Chongqing Academy of Chinese Materia Medica (jbky20200026 and jbky20210029), Chongqing Municipal Health Commission (Chongqing Traditional Chinese Medicine Inheritance and Innovation Team Construction Project, [2022] No. 33), and China Postdoctoral Science Foundation (No. 2021MD703919).

## Conflict of interest

The authors declare that the research was conducted in the absence of any commercial or financial relationships that could be construed as a potential conflict of interest.

## Publisher’s note

All claims expressed in this article are solely those of the authors and do not necessarily represent those of their affiliated organizations, or those of the publisher, the editors and the reviewers. Any product that may be evaluated in this article, or claim that may be made by its manufacturer, is not guaranteed or endorsed by the publisher.

## Footnotes

^1^ http://en.biocloud.net/
